# Supplementary material for: Empirical methods for controlling false positives and estimating confidence in ChIP-Seq peaks
Source: BMC Bioinformatics. 2008 Dec 5;9:523. doi: 10.1186/1471-2105-9-523 (PMC2628906; doi:10.1186/1471-2105-9-523)
Supplement: Additional File 1 — A variety of html documents from the USeq web site detailing the available applications, their best usage, output file type descriptions, command line menus, etc. [file 1471-2105-9-523-S1.zip › USeqUserGuides/JavaDoc.zip!/JavaDoc/edu/utah/seq/data/SmoothingWindow.html]

SmoothingWindow


|  |  |  |
| --- | --- | --- |
| **Overview** \| **Package** \| **Class** \| **Use** \| **Tree** \| **Deprecated** \| **Index** \| **Help** | |  |
| **PREV CLASS**   **NEXT CLASS** | **FRAMES**    **NO FRAMES**     **All Classes** |  |
| SUMMARY: NESTED \| FIELD \| CONSTR \| METHOD | DETAIL: FIELD \| CONSTR \| METHOD |  |


---


## edu.utah.seq.data Class SmoothingWindow

```
java.lang.Object
  edu.utah.seq.data.SmoothingWindow
```

**All Implemented Interfaces:**: java.io.Serializable

---

``` public class SmoothingWindow extends java.lang.Object implements java.io.Serializable ```

Coordinates are interbase!

**See Also:**: Serialized Form

---

| **Field Summary** | |
| --- | --- |
| `static long` | `serialVersionUID` |


**Constructor Summary**

`SmoothingWindow()`

`SmoothingWindow(int start,
int stop,
float[] scores)`


| **Method Summary** | |
| --- | --- |
| `float[]` | `getScores()` |
| `int` | `getStart()` |
| `int` | `getStop()` |
| `void` | `setScores(float[] scores)` |
| `void` | `setStart(int start)` |
| `void` | `setStop(int stop)` |

**Methods inherited from class java.lang.Object**

`equals, getClass, hashCode, notify, notifyAll, toString, wait, wait, wait`

**Field Detail**

### serialVersionUID

```
public static final long serialVersionUID
```

**See Also:**: Constant Field Values


**Constructor Detail**

### SmoothingWindow

```
public SmoothingWindow()
```

---


### SmoothingWindow

```
public SmoothingWindow(int start,
                       int stop,
                       float[] scores)
```


**Method Detail**

### getScores

```
public float[] getScores()
```

---


### setScores

```
public void setScores(float[] scores)
```

---


### getStart

```
public int getStart()
```

---


### setStart

```
public void setStart(int start)
```

---


### getStop

```
public int getStop()
```

---


### setStop

```
public void setStop(int stop)
```


---


|  |  |  |
| --- | --- | --- |
| **Overview** \| **Package** \| **Class** \| **Use** \| **Tree** \| **Deprecated** \| **Index** \| **Help** | |  |
| **PREV CLASS**   **NEXT CLASS** | **FRAMES**    **NO FRAMES**     **All Classes** |  |
| SUMMARY: NESTED \| FIELD \| CONSTR \| METHOD | DETAIL: FIELD \| CONSTR \| METHOD |  |


---
